# New Alpha-Amylase Inhibitory Metabolites from Pericarps of *Garcinia mangostana*

**DOI:** 10.3390/life12030384

**Published:** 2022-03-07

**Authors:** Nabil Abdulhafiz Alhakamy, Gamal Abdallah Mohamed, Usama Ahmed Fahmy, Basma Ghazi Eid, Osama Abdelhakim Aly Ahmed, Mohammed Wanees Al-Rabia, Amgad Ibrahim Mansour Khedr, Mohammed Zahid Nasrullah, Sabrin Ragab Mohamed Ibrahim

**Affiliations:** 1 Department of Pharmaceutics, Faculty of Pharmacy, King Abdulaziz University, Jeddah 21589, Saudi Arabia; uahmedkauedu.sa@kau.edu.sa (U.A.F.); oaahmed@kau.edu.sa (O.A.A.A.); 2 Center of Excellence for Drug Research and Pharmaceutical Industries, King Abdulaziz University, Jeddah 21589, Saudi Arabia; mnasrullah@kau.edu.sa; 3 Mohamed Saeed Tamer Chair for Pharmaceutical Industries, King Abdulaziz University, Jeddah 21589, Saudi Arabia; 4 Department of Natural Products and Alternative Medicine, Faculty of Pharmacy, King Abdulaziz University, Jeddah 21589, Saudi Arabia; 5 Department of Pharmacology and Toxicology, Faculty of Pharmacy, King Abdulaziz University, Jeddah 21589, Saudi Arabia; beid@kau.edu.sa; 6 Department of Medical Microbiology and Parasitology, Faculty of Medicine, King Abdulaziz University, Jeddah 21589, Saudi Arabia; mwalrabia@kau.edu.sa; 7 Health Promotion Center, King Abdulaziz University, Jeddah 21589, Saudi Arabia; 8 Department of Pharmacognosy, Faculty of Pharmacy, Port Said University, Port Said 42526, Egypt; amged.ibrahim@pharm.psu.edu.eg; 9 Department of Chemistry, Batterjee Medical College, Preparatory Year Program, Jeddah 21442, Saudi Arabia; sabrin.ibrahim@bmc.edu.sa; 10 Department of Pharmacognosy, Faculty of Pharmacy, Assiut University, Assiut 71526, Egypt

**Keywords:** benzophenones, *Garcinia mangostana*, Clusiaceae, amylase inhibitory, diabetes, docking study, molecular dynamics

## Abstract

Two new benzophenones: garcimangophenones A (**6**) and B (**7**) and five formerly reported metabolites were purified from the pericarps EtOAc fraction of *Garcinia mangostana* ((GM) Clusiaceae). Their structures were characterized by various spectral techniques and by comparing with the literature. The α-amylase inhibitory (AAI) potential of the isolated metabolites was assessed. Compounds **7** and **6** had significant AAI activity (IC_50_ 9.3 and 12.2 µM, respectively) compared with acarbose (IC_50_ 6.4 µM, reference α-amylase inhibitor). On the other hand, **5** had a moderate activity. Additionally, their activity towards the α-amylase was assessed utilizing docking studies and molecular dynamics (MD) simulations. The docking and predictive binding energy estimations were accomplished using reported crystal structure of the α-amylase (PDB ID: 5TD4). Compounds **7** and **6** possessed highly negative docking scores of −11.3 and −8.2 kcal/mol, when complexed with 5TD4, respectively while acarbose had a docking score of −16.1 kcal/mol, when complexed with 5TD4. By using molecular dynamics simulations, the compounds stability in the complexes with the α-amylase was analyzed, and it was found to be stable over the course of 50 ns. The results suggested that the benzophenone derivative **7** may be potential α-amylase inhibitors. However, further investigations to support these findings are required.

## 1. Introduction

Diabetes is a major severe health problem worldwide. Its treatment without any side effects is still a great challenge. It is characterized by chronic hyperglycemia with carbohydrate, protein, and lipid metabolic disturbances due to defects in insulin action and/or insulin secretion [1,2,3,4]. It is associated with various macrovascular (e.g., peripheral vascular and ischaemic heart diseases and stroke) and microvascular (e.g., nephropathy, neuropathy, and retinopathy) complications [5,6]. Fruits are considered as one of the major sources of bioactive secondary metabolites such as carotenoids, polyphenols, glucosinolates, sterols, and saponins, as well as vitamins and fibers [7]. It was reported that the consumption of whole fruits and vegetables reduces the risk for developing diabetes and diabetes-related complications [5,8]. Tropical fruits consumption has significantly increased due to growing knowledge of their health-promoting and nutritional properties [9]. One of the therapeutic strategies for diabetes management is the postprandial hyperglycemia reduction by hindering glucose absorption via suppressing carbohydrate-hydrolyzing enzymes such as α-glucosidase and α-amylase [10]. The α-amylase inhibition is singled out as an effective therapeutic target in treating type II diabetes and obesity [11,12]. Synthetic α-amylase inhibitors (AAIs) are reported to cause many critical side effects, such as liver disorders [13]. Nowadays, many researchers try to find a safe AAI with no or least side effects. Phenolic metabolites have been systematically proven to have a remarkable effect in reducing postprandial hyperglycemia and improving diabetes-associated complications [14].

**Figure 1:**
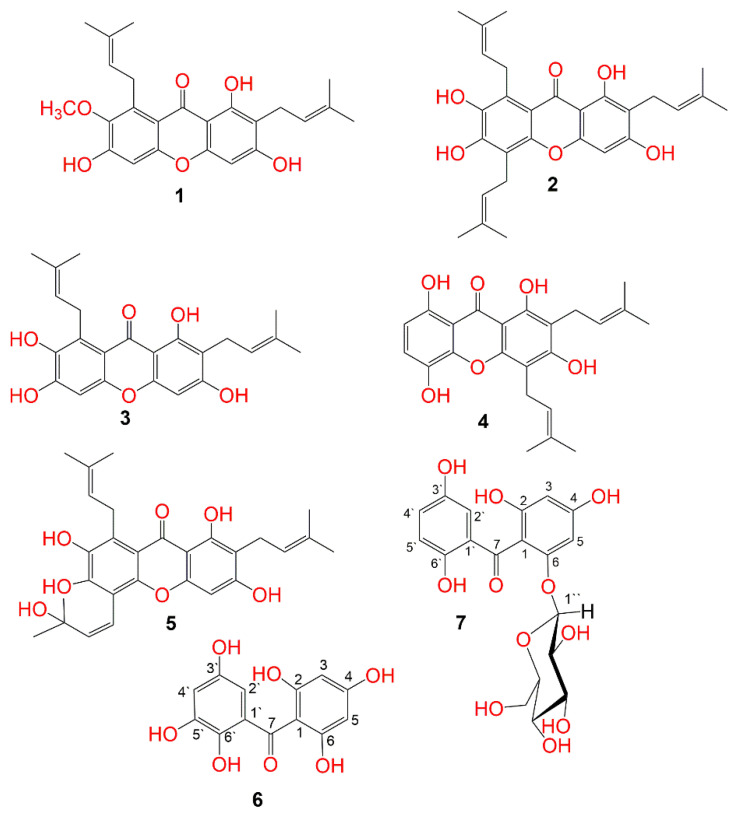
Structures of the isolated compounds (**1**–**7**).

Garcinia mangostana ((GM) purple mangosteen, mangosteen, mangkhut, queen of fruits) is a tropical fruit that is widely cultivated in Myanmar, Thailand, Vietnam, India, Cambodia, Malaysia, Netherlands Antilles, Sri Lanka, Philippines, Colombia, Central America, and tropical Africa [15]. This fruit has gained increasing acceptance due to its pleasant sweet-sour taste and distinctive aroma. It is also a rich source of nutrients and health-promoting phytochemicals, including carotenoids, ascorbic acid, oxygenated and prenylated xanthones, flavonoids, anthocyanins, phenolic acids, and benzophenones which possess a wide extent of bioactivities [16,17,18,19,20,21,22]. In Southeast Asia, it is used as a traditional medicine for treating dysentery, abdominal pain, suppuration, wound infections, and chronic ulcers [23,24,25]. Various in-vivo and in-vitro studies proved that GM pericarp extracts (GME) had substantial insulin-sensitizing and glucose-lowering potentials [26,27]. GME displayed a hypoglycemic potential via increasing the insulin-producing β-cells’ population, which was attributed to their antioxidant-phenolic metabolites [27]. Adnyana et al. reported that α-mangostin and the pericarp extract of GM had a concentration-dependent α-amylase inhibitory potential [28]. Moreover, GM bettered the STZ (streptozotocin)-produced impairment to β-cells and pancreatic glands through enhancing insulin production and amending the sensitivity to insulin decrease in diabetic mice [29]. Besides, it retarded the glucose absorption via suppressing the enzymes accountable for carbohydrates hydrolysis such as α-amylase and α-glucosidase [30]. Interestingly, many GM xanthones have been reported as α-glucosidase and α-amylase inhibitors as they lessened postprandial hyperglycemia by prohibiting the absorption of glucose [2,3,4,12,31]. A clinical study by Watanabe et al. reported that GME supplementation for 26 weeks resulted in improving glucose hemostasis and increasing insulin sensitization in obese insulin-resistant female patients [32]. Our previous studies of GM reported the isolation of xanthones, flavonoids, and phenolics with cytotoxic, vasodilatation, hepato-protective, antioxidant, and glycation end-product and α-amylase inhibitory capacities [18,19,20,24,25,31,33,34]. In continuing of our goal to discover structurally diverse bio-metabolites from GM, this work described the purification and structural elucidation of two new benzophenone derivatives, along with five known metabolites from its EtOAc-soluble extract (Figure 1). They were elucidated unambiguously by NMR and MS techniques, in addition to comparing their data with the known related compounds. Their α-amylase inhibition capacity was estimated. Additionally, docking studies and molecular dynamics (MDs) simulations towards α-amylase were carried out.

## 2. Materials and Methods

### 2.1. General Experimental Procedures

Spectrometer Hitachi-300, Shimadzu-400 Infrared, and digital polarimeter JASCO/DIP-370 were utilized for UV, IR, and optical rotation measurements. ESI- and HR-ESIMS measuring was performed on LCQ/DECA and Micromass-Qtof 2 mass spectrometer, respectively. NMR analyses were accomplished using the 400 and 850 BRUKER/AVANCE instrument. SiO_2_ 60, Sephadex LH-20, and RP-1 were used for chromatographic analysis. The purification was performed on a 6-mL solid-phase LiChrolut/RP-18 extraction tube.

### 2.2. Plant Material

The fruits were obtained from a Saudi local market in March 2019. Its verification was proven by Dr. Emad Al-Sharif (Faculty of Science and Arts, King Abdulaziz University) and a voucher (no. GM1424) specimen was maintained in the Faculty of Pharmacy`s herbarium, KAU.

### 2.3. Extraction and Separation

The dried pericarps (1.2 kg) were extracted using MeOH (4 L × 5, room temperature) until exhaustion and the resulted extracts were evaporated under-vacuum. The methanol extract (29 g, GMT) was mixed in distilled H_2_O (300 mL) and partitioned successively among *n*-hexane and EtOAc (500 mL × 5, each) to furnish 3.1, 7.5, 15.9 g *n*-hexane, EtOAc, and aqueous fractions, respectively. The EtOAc fraction (7.4 g) was submitted to silica gel column chromatography (SiO_2_ CC), eluting with EtOAC/*n*-hexane gradient to afford four subfractions: GME-1 (75:25), GME-2 (50:50), GME-3 (25:75), and GME-4 (100% EtOAc). Subfraction GME-2 (1.19 g) was separated on SiO_2_ CC using *n*-hexane:EtOAc gradient to obtain five fractions GME-2A to GME-2E. Fraction GME-2A (274 mg) was chromatographed on SiO_2_ CC, eluting with EtOAC/*n*-hexane gradient to obtain **1** (189 mg). Fraction GME-2B (191 mg) was treated similar to GME-2A to obtain **2** (15 mg). Fraction GME-2C (378 mg) was treated similar to GME-2B to afford **3** and **4** that were purified on RP-18 column (100 g, 50 × 3 cm) using H_2_O/MeOH gradient to yield **3** (17 mg) and **4** (31 mg). Subfraction GME-3 (2.19 g) was subjected to SiO_2_ CC (200 g × 50 × 3 cm) using MeOH/CHCl_3_ gradient to obtain seven fractions; GME-3A to GME-3G. Fraction GME-3B (120 mg) was subjected to SiO_2_ CC using MeOH/CHCl_3_ gradient, RP-18-LiChrolut extraction tube (acetonitrile:H_2_O gradient) to furnish **5** (9.5 mg). Fractions GME-3C to GME-3E (746 mg) were gathered based on TLC plate, their Sephadex LH-20 (50 g, 50 × 3 cm) using MeOH yielded **6** and **7**. Their purification was achieved on RP-18 CC, using H_2_O:MeOH gradient to obtain **6** (11.2 mg) and **7** (15.7 mg).

#### 2.3.1. Garcimangophenone A (**6**)

Light yellow powder; UV (MeOH) λ*_max_* (log *ε*): 206 (4.31), 223 sh, 296 (3.79), 318 (3.09) nm; IR (KBr) γ*_max_*: 3317, 1623, 1601, 1589 cm^−1^; HRESIMS: *m*/*z* 279.0515 [M+H]^+^ (calcd for C_13_H_11_O_7_, 279.0505), ^1^H and ^13^C NMR (Table 1).

#### 2.3.2. Garcimangophenone B (**7**)

Brown amorphous powder; [α]_D_ +26.8 (c 0.2, MeOH); IR (KBr) γ*_max_*: 3342, 1632, 1608, 1578 cm^−1^; UV (MeOH) λ*_max_* (log *ε*): 209 (4.42), 220 sh, 286 (3.92), 308 (3.24); HRESIMS: *m/z* 425.1078 [M+H]^+^ (calcd for 425.1084 for C_19_H_21_O_11_); ^1^H NMR and ^13^C NMR (Table 1).

### 2.4. α-Amylase Inhibition

The new benzophenone derivatives (**6** and **7**) and the known xanthone (**5**) were evaluated for their AAI potential at concentrations of 5, 10, 20, and 40 µM [2,3,4,35,36]. The method is based on the assay of α-amylase using EnzChek^®^ Ultra-Amylase Assay Kit (E33651) (Thermo-Fisher Scientific Inc., Waltham, MA, USA). The provided stock solution of dye quenching (DQ^TM^) starch and porcine pancreatic α-amylase enzyme (Sigma-Aldrich, Hamburg, Germany) were diluted with the reaction buffer (pH 6.9) according to the reported protocol [36]. To the microplate wells, the tested compound (10 µL) in DMSO, diluted enzyme (50 µL), and 40 µL of the reaction buffer were added and allowed to stand at room temperature for 5 min, then DQ^TM^ starch (100 µL) was added. The fluorescence intensity of the digestion products from the DQ^TM^ starch (with or without compounds) was measured using a Tecan Genios microplate reader at λ*_max_* 485 ± 10 nm starting from zero min to 60 min at 10 min intervals. All experiments were performed in triplicate. Acarbose was utilized as positive control. The IC_50_ values were calculated by linear regression analysis [2,3,4,35,36].

### 2.5. Molecular Docking and Dynamics Studies

#### 2.5.1. Preparation of PDB Structures

The human pancreatic α-amylase (5TD4) was selected for docking studies. It was reported that the molecular models for the human and porcine pancreatic α-amylases are extremely similar [37,38,39]. The structure of the target, 5TD4, was downloaded from the Protein Data Bank and prepared and optimized using the “Protein preparation wizard” tool of the Schrödinger suite [40]. Some modifications were performed for optimization such as breaking the bonds to metals, adding zero-order bonds between metals and close atoms, and correcting charges. LigPrep was also used for preparing ligands in which a pH of 7.0 ± 2.0 was used for the generation of cofactors and metals het states. In addition, PROPKA was used for enhancing hydrogen bonds at pH 7, water molecules more than 3 Å from HET groups were extracted, and OSPL4 field was used for restrained minimization.

#### 2.5.2. Receptor Grids Generation and Docking

Grid generation and the docking of ligands were both performed using Glide [41]. The grid was generated by using the crystal structure (PDB ID: 5TD4). For the PDB 5TD4, the binding region was established by selecting the native inhibitor T163. The nonpolar atoms were set for Van der Waals radii scaling factor to 1, and the partial charge cut-off was 0.25. The docking of ligands was performed using the “ligand docking” tool of Schrödinger suite. The protocol was extra precision (XP) and all the other settings were kept in their default form.

#### 2.5.3. MD Simulation of Compound **7** and Acarbose in Complex with 5TD4

The Schrödinger suite [42,43] was used to run the molecular dynamic simulation. The TIP3P solvent was selected, and an orthorhombic shape box was chosen. A side distance box was set to 10 Å, and Na^+^ ions were added for the system neutralization. The molecular dynamic calculations were continued for 50 ns per trajectory, and the number of atoms, pressure, and temperature were kept constant throughout the simulation.

## 3. Results and Discussion

### 3.1. Purification of Compounds

The MeOH extract was suspended in H_2_O and partitioned between *n*-hexane and EtOAc. The EtOAc fraction was successively treated utilizing Sephadex, SiO_2,_ and RP-18 columns to provide two new (**6** and **7**) and five known metabolites (**1–5**) (Figure 1). These metabolites were identified based on the spectral analyses and comparison with literature.

### 3.2. Structural Assignment of Compounds ***6*** and ***7***

**Figure 2:**
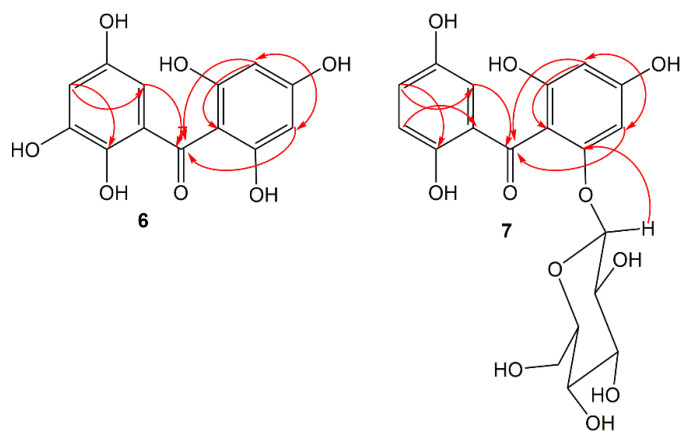
Some key HMBC correlations of compounds **6** and **7**.

Compound **6** was separated as a light-yellow powder and provided an FeCl_3_ positive test, revealing its phenolic nature. Its HRESIMS demonstrated a pseudo-molecular peak at *m*/*z* 279.0515 [M+H]^+^ (calcd for C_13_H_11_O_7_, 279.0505), consistent with a molecular formula C_13_H_10_O_7_. This formula required nine degrees of unsaturation. The UV revealed bands at 206, 296, and 318 nm, suggesting **6** to have a benzophenone skeleton [44]. Characteristic bands at 3317, 1623, and 1601 and 1589 cm^−1^ for chelated OH, carbonyl, and aromatic C=C functionalities, respectively were observed in the IR spectrum [45]. The ^13^C and HSQC spectra showed 13 carbon resonances, comprising four aromatic methines and nine quaternary carbons, including one carbonyl at δ_C_ 200.6 (C-7) and six for oxygen-linked carbons (Table 1). In the ^1^H NMR of **6**, the appearance of an aromatic signal at δ_H_ 5.83 (2H, brs, H-3, 5), relating with the carbon at δ_C_ 95.8 (C-3, 5) in the HSQC suggested the presence of a symmetrically substituted phloroglucinol ring (ring A) in **6** [45]. This was confirmed by the HMBC-cross-peaks of H-3/C-2, C-1, C-4, and C-5 and H-5/C-1, C-3, C-4, and C-6. Moreover, the ^1^H NMR signals at δ_H_ 6.52 (d, J = 2.4 Hz, H-2`) and 6.36 (d, J = 2.4 Hz, H-4`) were attributed to two *m*-coupled aromatic protons. They had HSQC-cross-peaks to the carbons at δ_C_ 107.4 (C-2`) and 112.1 (C-4`). This was consistent with the existence of a hydroxyquinol moiety (1,2,3,5-tetrasubstituted phenyl, ring B). In the HMBC, the cross-peaks of H-2`/C-4` and C-6` and H-4`/C-2`, C-5`, and C-6` assured this moiety (Figure 2). The link of rings A and B via the carbonyl group to provide the benzophenone core was established based on HMBC correlations from H-3 and H-5/C-7 and H-2`/C-7 and C-1. Based on the fore-mentioned evidences, the structure of **6** was elucidated and named garcimangophenone A.

Compound **7** was separated as an amorphous brown powder. It displayed bands at 3342 (OH), 1632 (C=O), and 1608 and 1578 (C=C) cm^−1^ in the IR and at 209, 286, and 308 nm, respectively, in the UV spectrum. It had a molecular formula C_19_H_20_O_11_, which was specified from the observed pseudo-molecular ion peak in HRESIMS at m/z 425.1078 (calcd for 425.1084 for C_19_H_21_O_11_), requiring ten degrees of unsaturation. A characteristic fragment ion peak at *m*/*z* 262.0433 [M+H-hexose]^+^ was observed in the HRESIMS, indicating **7** had a hexose moiety. In the ^13^C and HSQC, 19 carbon signals were observed, including one methylene, ten methines, and eight quaternary carbons five of them for oxygenated aromatic carbons at 163.8 (C-2), 161.7 (C-4), 159.5 (C-6), 154.2 (C-3`), and 155.8 (C-6`). Compound **7** had one degree of unsaturation and 146 mass units more than **6**. Its NMR spectral data were comparable with those of **6**, except for the 1,2,3,5-tetrasubstituted benzene unit in **6** that had been replaced by 1,2,5-trisubstituted benzene unit in **7**. In ^1^H and ^13^C NMR signals for a symmetrically-substituted phloroglucinol (ring A) at δ_H_ 6.06 (d, J = 2.4 Hz, H-3)/δ_C_ 98.1 (C-3) and 6.21 (d, J = 2.4 Hz, H-5)/δ_C_ 95.7 (C-5) were observed, which was further assured by the HMBC-cross-peaks of H-3/C-1, C-2, C-4, and C-5 and H-5/C-1, C-3, and C-4. Additionally, a doublet signal at δ_H_ 4.88 (J = 7.6 Hz, H-1``) for an anomeric proton, having HSQC cross-peak to the carbon at δ_C_ 101.6 characterized the β-D-glucopyranosyl unit in **7** [46]. The other sugar signals at δ_H_ 2.92 (H-1``)/δ_C_ 74.6 (C-1``), 3.30 (H-3``)/δ_C_ 77.8 (C-3``), 3.29 (H-4``)/δ_C_ 71.0 (C-4``), 3.32 (H-5``)/δ_C_ 78.2 (C-5``), and 3.86 and 3.69 (H-6``)/δ_C_ 62.5 (C-6``) were observed in the ^1^H and ^13^C NMR spectra. Further, the HMB-cross-peaks of H-3``and H-2``/C-1``, H-4``/C-2`` and C-6``, H-5``/C-3`` and C-6``, and H-6``/C-4`` and C-5`` established the β-D-glucopyranosyl unit. The HMBC cross-peak of H-1`` to C-6 (δ_C_ 159.5) indicated the connectivity of glucose moiety at C-6 of ring A. Furthermore, ^1^H NMR spectrum also displayed three coupled aromatic protons, resonating at δ_H_ 6.96 (H-2`), 7.18 (H-4`), and 7.22 (H-5`). They had HSQC cross-peaks to the carbons at δ_C_ 114.2, 121.4, and 120.4, respectively, which characterized the existence of a hydroquinone moiety in **7** (ring B) [44,45]. This was further secured based on the observed HMBC cross-peaks of H-5`/C-1` and C-3`, H-4`/C-2` and C-6`, and H-2`/C-4` and C-6` (Figure 2). The connectivity of the carbonyl group (δ_C_ 199.3, C-7) at C-1 and C-1` of rings A and B, respectively, was established by the HMBC correlations of H-3, H-5, and H-2` to C-7. Therefore, the structure of **7** was established as depicted and named garcimangophenone B.

### 3.3. Identification of Known Metabolites

The known metabolites were specified as *α*-mangostin (**1**) [15,47], garcinone E (**2**) [48], nor-mangostin (**3**) [49], gartanin (**4**) [50], and mangostanaxanthone VI (**5**) [23], by comparing their spectral data to the formerly published data of related metabolites.

### 3.4. α-Amylase Inhibitory Activity

Benzophenones are known to have diverse bioactivities such as antioxidant, antibacterial, cytotoxic, antitrypanosomal, and leishmanicidal activities [51,52,53,54]. However, there are limited reports regarding the AAI potential of Garcinia’s benzophenones. Therefore, the AAI potential of the new benzophenone derivatives (**6** and **7**), along with compound **5** was assessed. The results showed that compounds **7** and **6** demonstrated noticeable AAI potential (IC_50_ 9.3 and 12.2 µM, respectively), compared with acarbose (IC_50_ 6.4 µM). These results are in good agreement with the previous study by Akoro et al. that reported the potent AAI effect of gakolanone, a benzophenone derivative reported from G. kola [55]. On the other hand, **5** displayed moderate activity with an AAI (IC_50_ 16.1 µM). It is noteworthy that previous studies revealed the AAI potential of **1**–**4** [2,3,4].

### 3.5. Molecular Docking and Dynamics Studies

The hydrolysis of the glycosidic linkage in starch is catalyzed by the α-amylase enzyme [56]. The α-amylase active site consists of five major subsites that are necessary for the binding of longer substrates [57]. Three residues in the active site, Asp 300, Asp 197, and Glu 233 are important for the catalysis. The catalytic nucleophile, Asp 197, forms a covalent bond with the glucosyl in the S-1 subsite. The Glu233 is the acid-base catalyst as it protonates the leaving group and deprotonates the water. The Glu300 is also essential for positioning the catalytic water for hydrolysis [58].

#### 3.5.1. Preparations of Ligands and Proteins

Converting 2D structures to 3D structures, tautomerization, and ionization were all performed using LigPrep and resulted in the generation of 121 minimized structures. The 5TD4 was prepared and optimized by the Protein Preparation Wizard. Optimization included the H-bonding and minimization of the geometry, in addition to assigning the appropriate charges and force field treatment.

#### 3.5.2. Molecular Docking Studies

**Figure 3:**
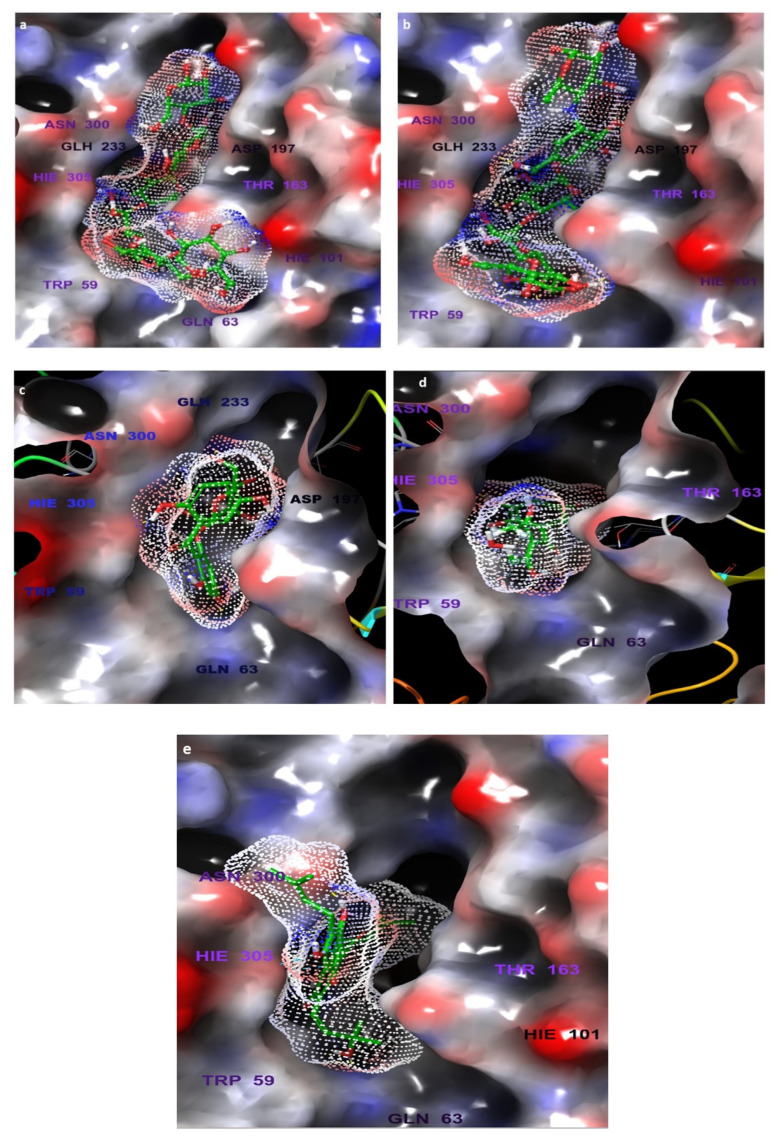
The α-amylase prepared via Protein Preparation Wizard, Maestro. H-bonding network optimized, and geometry minimized structures of α-amylase PDB ID: 5TD4 complexed with (**a**) native inhibitor T163, (**b**) acarbose, (**c**) compound **7**, (**d**) compound **6**, and (**e**) compound **5**.

The Receptor Grid Generation tool of Glide in Maestro was used for defining the grid box with the prepared α-amylase and then the 3D molecular structures (native inhibitor T163, acarbose, **7**, **6**, and **5**) were docked with the 5TD4 binding site (Figure 3) and the docking scores were reported (Table 2). The scores are an indication of the most strongly bound ligand and binding affinities. The T163 exhibited the highest docking score, followed by acarbose, **7**, **6**, and **5** which were −19.081, −16.078, −11.297, −8.185, and −6.726 complexed with 5TD4, respectively.

**Figure 4:**
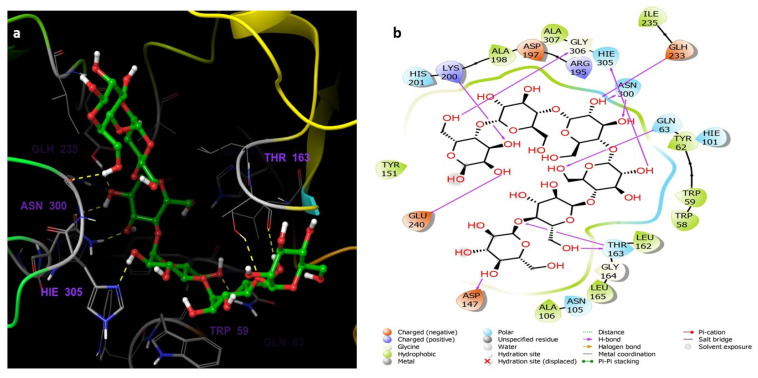
(**a**) The inter-molecular contact analysis of protein-ligand complexes and putative binding mode of native inhibitor T163 in the binding site of α-amylase PDB: 5TD4. Native inhibitor is represented as green sticks. The binding site amino acids residues are represented as grey sticks, and H-bonds are represented in yellow dashed lines, (**b**) 2D depiction of the ligand-protein interactions.

The docking analysis was performed to study the possible binding interactions between the different structures and the α-amylase (PDB ID:5TD4). The analysis of the docking between T163, the native inhibitor, and 5TD4 revealed various hydrogen bonds with the amino acid residues, most important are the hydrogen bonds with Asp197 and Glh233 which are the catalytic amino acids (Figure 4). 

**Figure 5:**
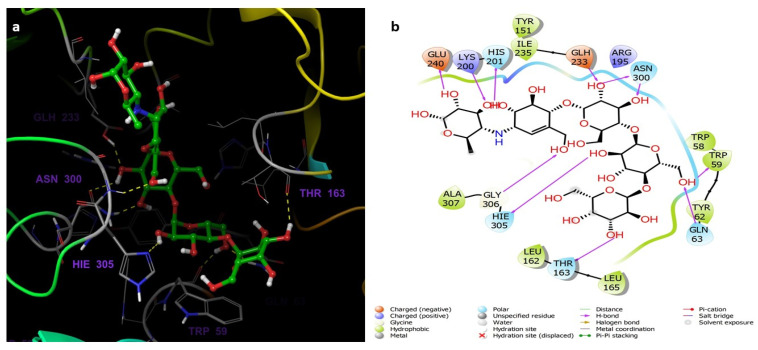
(**a**) The inter-molecular contact analysis of protein-ligand complexes and putative binding mode of acarbose in the binding site of α = amylase PDB: 5TD4. Acarbose is represented as green sticks. The binding site amino acids residues are represented as grey sticks, and H-bonds are represented in yellow dashed lines, (**b**) 2D depiction of the ligand-protein interactions.

Acarbose complexed with 5TD4 also showed hydrogen bonds with the catalytic amino acid residues (Figure 5).

**Figure 6:**
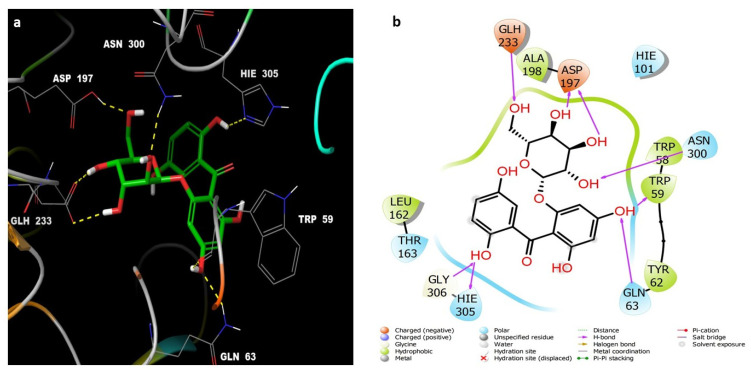
(**a**) The inter-molecular contact analysis of protein-ligand complexes and putative binding mode of compound **7** in the binding site of α-amylase PDB: 5TD4. Compound **7** is shown as green sticks. The binding site amino acids residues are represented as grey sticks, and H-bonds are represented in yellow dashed lines, (**b**) 2D depiction of the ligand-protein interactions.

**Figure 7:**
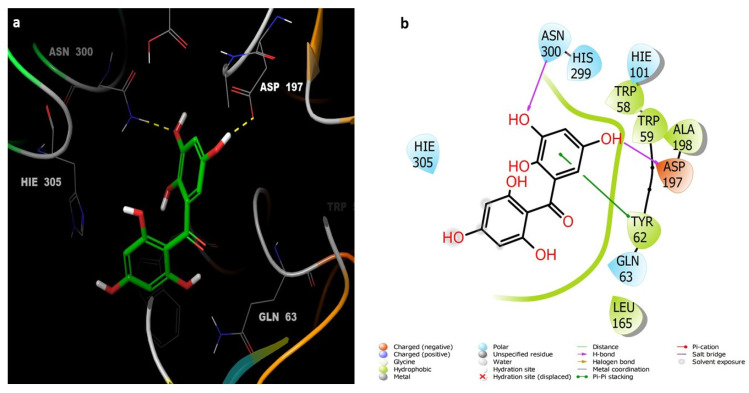
(**a**) The inter-molecular contact analysis of protein-ligand complexes and putative binding mode of compound **6** in the binding site of α-amylase PDB: 5TD4. Compound **6** is shown as green sticks. The binding site amino acids residues are represented as grey sticks, and H-bonds are represented in yellow dashed lines, (**b**) 2D depiction of the ligand-protein interactions.

**Figure 8:**
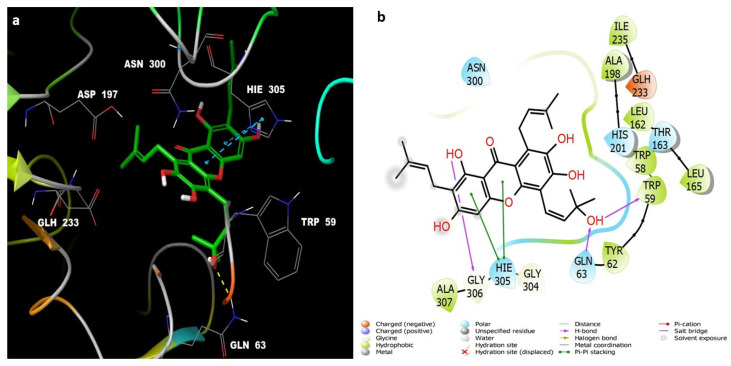
(**a**) The inter-molecular contact analysis of protein-ligand complexes and putative binding mode of compound **5** in the binding site of α-amylase PDB: 5TD4. Compound **5** is presented as green sticks. The binding site amino acids residues are represented as grey sticks, and H-bonds are represented in yellow dashed lines, (**b**) 2D depiction of the ligand-protein interactions.

Compounds **7** (Figure 6) and **6** (Figure 7) also showed similar binding interactions; however, compound **5** (Figure 8) lacked some of the major binding interactions which decreased its activity.

#### 3.5.3. Molecular Dynamic Simulation

The movement of atoms with respect to time is computed through a molecular dynamics (MDs) simulation. The dynamics of the different atoms and the conformational stability both play important roles in the functioning of the essential biological macromolecules such as receptors and proteins [59]. For this reason, different properties of the systems such as root mean square deviation (RSMD), Cα-root mean square fluctuations (RMSF), the gyration radius (Rg), and location of inter-molecular H-bonds were studied in order to determine the dynamics of the system. The molecular docking predicts the binding mode of ligands to receptors which is a good starting point to study the stability of interactions. The Desmond software was used to study the frequency and stability of compound **7** and acarbose complexed with the α-amylase protein (PDB ID:5TD4). Two MD simulations were run for the complexes for 50 ns, and the complexes’ structures were fixed at a pH of 7.0 ± 2.0. The stability of the complexes was tested by analyzing the interaction map and the root mean square deviation plot of the ligand and the protein.

**Figure 9:**
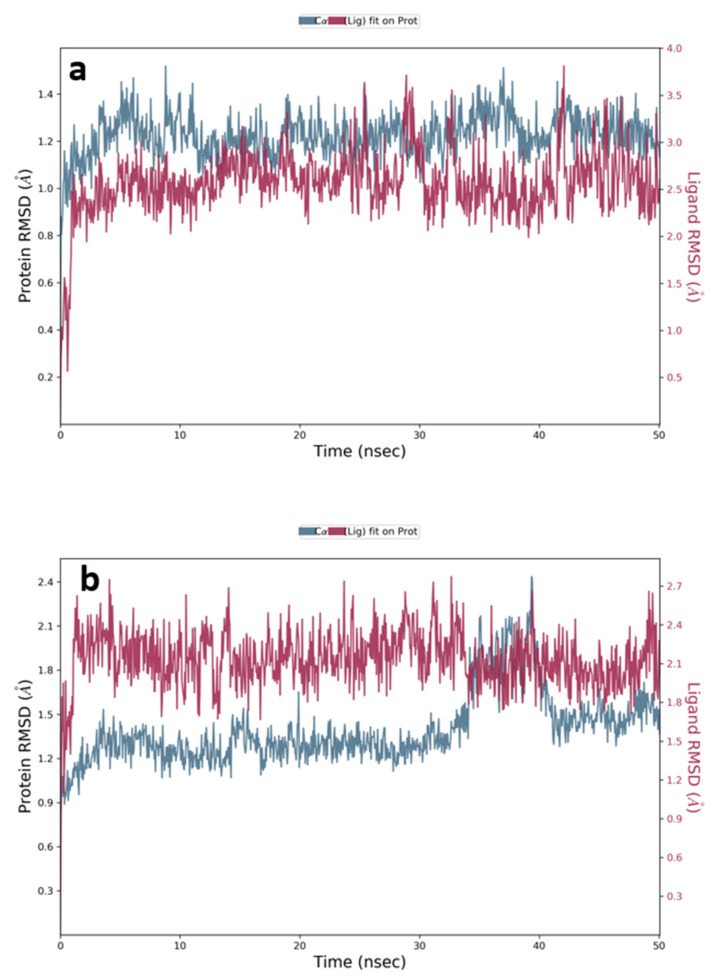
RMSD analysis for (**a**) compound **7** and (**b**) acarbose complexed with α-amylase (PDB Code: 5TD4) of MD simulation trajectory. The 50 ns simulation time asserts the stability of the complex without any changes in the structure.

Figure 9a,b represent the plots of the RMSD for the α-amylase (PDB ID: 5TD4) complexed with compound **7** and acarbose, respectively. Looking at the RMSD values, it is clear that the acarbose was stabilized with the protein at about 2 Å after 35 ns and remained stable until 50 ns. However, the plot shows that the complex with compound **7** remained stable during the whole simulation time (50 ns) in relation to the reference time (0 ns). Although the plots showed some fluctuations at the time of simulation, all fluctuations are considered non-significant, as they were within an acceptable range between 1 and 3 Å.

**Figure 10:**
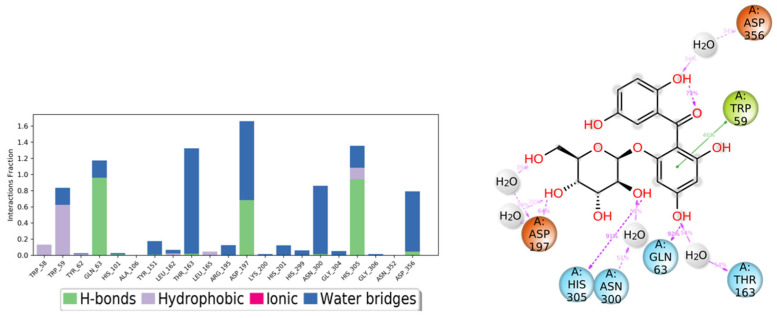
The α-amylase binding interactions with compound **7** throughout the simulation. The major interactions that occurred are Hydrogen bonds, water bridges, and hydrophobic interaction.

The binding interactions between compound **7** and the residues of the α-amylase (PDB ID:5TD4) are illustrated in Figure 10 which shows several interactions with residues Asp197 (one of the catalytic amino acids), His305, Thr163, Gln63, Asn300, Asp356, and Trp59. The interactions are mostly hydrogen bonds and water bridges with a few hydrophobic interactions. The amino acid residue Asp197 interacts with the ligand through a hydrogen bond directly or a hydrogen bond mediated by a water molecule which were retained for more than 100% of the simulation time. The amino acid residue His305 interacts with the ligand through three types of interactions: H bond, H bond mediated by a water molecule, and a hydrophobic interaction during the simulation. Gln63 forms a hydrogen bond with the ligand which is retained for 90% of the simulation time in addition to a hydrogen bond through a water bridge. Hydrophobic interaction between Trp59 and an aromatic ring in the ligand was maintained for 60% of the 50 ns simulation time.

**Figure 11:**
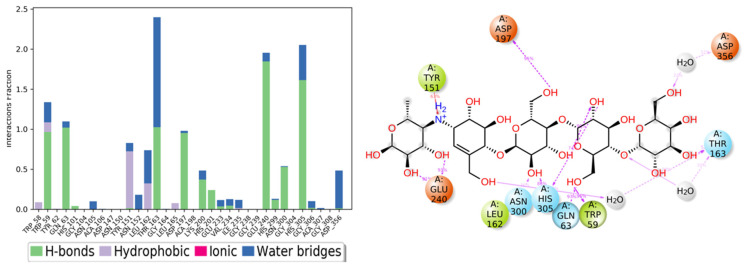
The α-amylase binding interactions with acarbose throughout the simulation. The major interactions that occurred are Hydrogen bonds, water bridges, and hydrophobic interaction.

The detailed schematic diagram of binding interactions between acarbose and the residues of the α-amylase (PDB ID:5TD4) are illustrated in Figure 11. The docked poses were controlled through the simulation time of 50 ns, i.e., molecular interactions with residues Thr163, His305, Glu240, Trp59, Gln63, Asp197, Tyr151, Leu162, and Asn300 were observed. Figure 11 shows the ligand-protein interactions classified into different types: ionic, hydrophobic, hydrogen bonds, and water bridges. The interactions are mostly hydrogen bonds, which play a significant role in ligand binding. Most of the interactions remained for more than 100% of the simulation time which indicates multiple contacts of the same subtype with the ligand.

## 4. Conclusions

Two new benzophenones and five known metabolites were purified from the EtOAc-soluble fraction of GM pericarps. They were characterized by various spectral techniques. Compounds **6** and **7** displayed AAI activity. They also exhibited highly negative docking scores, when complexed with 5TD4. Their complexes with the α-amylase were found to be stable over the course of 50 ns. These results supported the previous reports that GM can potentially represent an appealing treatment of diabetes and its related disorders.

## Figures and Tables

**Table 1:** NMR spectral data of compounds **6** and **7** (CD_3_OD, 400 and 100 Hz).

No.	6			7		
	δ_H_ (m, *J* in Hz)	δ_C_ m	HMBC	δ_H_ (m, *J* in Hz)	δ_C_ m	HMBC
**1**	-	106.3 C	-	-	109.4 C	-
**2**	-	165.4 C	-	-	163.8 C	-
**3**	5.83 brs	95.8 CH	1, 2, 4, 5, 7	6.06 d (2.4)	98.1 CH	1, 2, 4, 5, 7
**4**	-	165.4 C	-	-	161.7 C	-
**5**	5.83 brs	95.8 CH	1, 3, 4, 6, 7	6.21 d (2.4)	95.7 CH	1, 3, 4, 7
**6**	-	165.5 C	-	-	159.5 C	-
**7**	-	200.6 C	-	-	199.3 C	-
**1`**	-	123.9 C	-	-	119.2 C	-
**2`**	6.52 d (2.4)	107.4 CH	4`, 6`, 1, 7	6.96 d (2.4)	114.2 CH	1`, 4`, 6`, 1, 7
**3`**	-	153.8 C	-	-	154.2 C	-
**4`**	6.36 d (2.4)	112.1 CH	2`, 5`, 6`	7.18 brd (8.0)	121.4 CH	2`, 5`, 6`
**5`**	-	148.8 C	-	7.22 d (8.0)	120.4 CH	1`, 3`, 4`, 6`
**6`**	-	145.7 C	-	-	155.8 C	-
**1``**	-	-	-	4.88 d (7.6)	101.6 CH	6
**2``**	-	-	-	2.92 m	74.6 CH	1``
**3``**	-	-	-	3.30 m	77.8 CH	1``
**4``**	-	-	-	3.29 m	71.0 CH	2``, 6``
**5``**	-	-	-	3.32 m	78.2 CH	3``, 6``
**6``**	-	-	-	3.86 dd (12.4, 2.0) 3.69 dd (12.4, 5.6)	62.5 CH_2_	4``, 5``

**Table 2:** In silico screening results of the different compounds against α-amylase (PDB: 5TD4).

Compd	Docking Score	XP GScore	Glide GScore	Glide eModel
Native_ Inhibitor_5TD4	−19.081	−19.081	−19.081	−104.865
Acarbose	−16.078	−16.641	−16.641	−115.364
**7**	−11.297	−11.46	−11.46	−75.209
**6**	−8.185	−8.596	−8.596	−50.318
**5**	−6.726	−6.824	−6.824	−67.078

## Data Availability

Not applicable.

## References

[1] Ibrahim S.R.M., Mohamed G.A., Zayed M.F., Ross S.A. (2017). 8-Hydroxyirilone 5-methyl ether and 8-hydroxyirilone, new antioxidant and α-amylase inhibitors isoflavonoids from Iris germanica rhizomes. Bioorg. Chem..

[2] Ibrahim S.R.M., Mohamed G.A., Khayat M.T., Ahmed S., Abo-Haded H., Alshali K.Z. (2019). Mangostanaxanthone VIIII, a new xanthone from *Garcinia mangostana* pericarps, α-amylase inhibitory activity, and molecular docking studies. Rev. Bras. Farmacogn..

[3] Ibrahim S.R.M., Mohamed G.A., Khayat M.T., Ahmed S., Abo-Haded H. (2019). Garcixanthone D, a new xanthone, and other xanthone derivatives from *Garcinia mangostana* pericarps: Their α-amylase inhibitory potential and molecular docking studies. Starch-Stärke.

[4] Ibrahim S.R.M., Mohamed G.A., Khayat M.T., Ahmed S., Abo-Haded H. (2019). α-Amylase inhibitors xanthones from *Garcinia mangostana* pericarps and its possible use for the treatment of diabetes with their molecular docking studies. J. Food. Biochem..

[5] Du H., Li L., Bennett D., Guo Y., Turnbull I., Yang L., Bragg F., Bian Z., Chen Y., Chen J. (2017). Fresh fruit consumption in relation to incident diabetes and diabetic vascular complications: A 7-y prospective study of 0.5 million Chinese adults. PLoS Med..

[6] Holman R.R., Paul S.K., Bethel M.A., Matthews D.R., Neil H.A. (2008). 10-year follow-up of intensive glucose control in type 2 diabetes. N. Engl. J. Med..

[7] Alothman M., Bhat R., Karim A.A. (2009). Antioxidant capacity and phenolic content of selected tropical fruits from Malaysia, extracted with different solvents. Food Chem..

[8] Muraki I., Imamura F., Manson J.E., Hu F.B., Willett W.C., van Dam R.M., Sun Q. (2013). Fruit consumption and risk of type 2 diabetes: Results from three prospective longitudinal cohort studies. BMJ.

[9] Ayala-Zavala J.F., Vega-Vega V., Rosas-Domínguez C., Palafox-Carlos H., Villa-Rodríguez J.A., Wasim Siddiqui M.D., González-Aguilar G.A. (2011). Agro-industrial potential of exotic fruit by-products as a source of food additives. Food Res. Int..

[10] Tundis R., Loizzo M.R., Menichini F. (2010). Natural products as alpha-amylase and alpha-glucosidase inhibitors and their hypoglycaemic potential in the treatment of diabetes: An update. Mini-Rev. Med. Chem..

[11] Sales P.M., Souza P.M., Simeoni L.A., Magalhães P.O., Silveira D. (2012). α-Amylase inhibitors: A review of raw material and isolated compounds from plant source. J. Pharm. Pharmaceut. Sci..

[12] Ryu H.W., Cho J.K., Curtis-Long M.J., Yuk H.J., Kim Y.S., Jung S., Kim Y.S., Lee B.W., Park K.H. (2011). α-Glucosidase inhibition and antihyperglycemic activity of prenylated xanthones from Garcinia mangostana. Phytochemistry.

[13] Asgar A. (2013). Anti-diabetic potential of phenolic compounds: A Review. Int. J. Food Prop..

[14] Serina J.J.C., Castilho P.C.M.F. (2021). Using polyphenols as a relevant therapy to diabetes and its complications, a review. Critical Rev. Food Sci. Nutr..

[15] Mohamed G.A., Ibrahim S.R.M., Shaaban M.I.A., Ross S.A. (2014). Mangostanaxanthones I and II, new xanthones from the pericarp of Garcinia mangostana. Fitoterapia.

[16] Palakawong C., Delaquis P. (2018). Mangosteen processing: A review. J. Food Process. Preserv..

[17] Abdallah H.M., El-Bassossy H., Mohamed G.A., El-Halawany A.M., Alshali K.Z., Banjar Z.M. (2016). Phenolics from Garcinia mangostana inhibit advanced glycation endproducts formation: Effect on amadori products, cross-linked structures and protein thiols. Molecules.

[18] Abdallah H.M., El-Bassossy H., Mohamed G.A., El-Halawany A.M., Alshali K.Z., Banjar Z.M. (2016). Phenolics from Garcinia mangostana alleviate exaggerated vasoconstriction in metabolic syndrome through direct vasodilatation and nitric oxide generation. BMC Complement. Altern. Med..

[19] Abdallah H.M., El-Bassossy H., Mohamed G.A., El-Halawany A.M., Alshali K.Z., Banjar Z.M. (2017). Mangostanaxanthones III and IV: Advanced glycation endproduct inhibitors from the pericarp of *Garcinia mangostana*. J. Nat. Med..

[20] Muchtaridi M., Puteri N.A., Milanda T., Musfiroh I. (2017). Validation analysis methods of α-mangostin, γ-mangostin and gartanin mixture in mangosteen (*Garcinia mangostana* L.) fruit rind extract from West Java with HPLC. J. App. Pharm. Sci..

[21] Jiang H.Z., Quan X.F., Tian W.X., Hu J.M., Wang P.C., Huang S.Z., Cheng Z.Q., Liang W.J., Zhou J., Ma X.F. (2010). Fatty acid synthase inhibitors of phenolic constituents isolated from *Garcinia mangostana*. Bioorg. Med. Chem. Lett..

[22] Zadernowski R., Czaplicki S., Naczk M. (2009). Phenolic acid profiles of mangosteen fruits (*Garcinia mangostana*). Food Chem..

[23] Mohamed G.A., Al-Abd A.M., El-Halawany A.M., Abdallaha H.M., Ibrahim S.R.M. (2017). New xanthones and cytotoxic constituents from *Garcinia mangostana* fruit hulls against human hepatocellular, breast, and colorectal cancer cell lines. J. Ethnopharmacol..

[24] Ibrahim S.R.M., Abdallah H.M., El-Halawany A.M., Radwan M.F., Shehata I.A., Al-Harshany E.M., Zayed M.F., Mohamed G.A. (2018). Garcixanthones B and C, new xanthones from the pericarps of Garcinia mangostana and their cytotoxic activity. Phytochem, Lett..

[25] Ibrahim S.R.M., Mohamed G.A., Elfaky M.A., Zayed M.F., El-Kholy A.A., Abdelmageed O.H., Ross S.A. (2018). Mangostanaxanthone VII, a new cytotoxic xanthone from *Garcinia mangostana*. Z. Naturforsch. C.

[26] Mekseepralard C., Areebambud C., Suksamrarn S., Jariyapongskul A. (2015). Effects of long-term alpha-mangostin supplementation on hyperglycemia and insulin resistance in type 2 diabetic rats induced by high fat diet and low dose streptozotocin. J. Med. Assoc. Thail..

[27] Taher M., Zakaria T.M.F.S.T., Susanti D., Zakaria Z.A. (2016). Hypoglycaemic activity of ethanolic extract of *Garcinia mangostana* Linn. in normoglycaemic and streptozotocin-induced diabetic rats. BMC Complement. Altern. Med..

[28] Adnyana K., Abuzaid A.S., Iskandar E.Y., Kurniati N.F. (2016). Pancreatic lipase and α-amylase inhibitory potential of mangosteen (*Garcinia mangostana* linn.) pericarp extract. Int. J. Med. Res. Health Sci..

[29] Husen S.A., Kalqutny S.H., Ansori A.N.M., Susilo R.J.K., Alymahdy A.D., Winarni D. (2017). Antioxidant and antidiabetic activity of *Garcinia mangostana* L. pericarp extract in streptozotocin-induced diabetic mice. Biosc. Res..

[30] Manaharan T., Palanisamy U.D., Ming C.H. (2012). Tropical plant extracts as potential antihyperglycemic agents. Molecules.

[31] Ibrahim S.R.M., Abdallah H.M., El-Halawany A.M., Nafady A.M., Mohamed G.A. (2019). Mangostanaxanthone VIII, a new xanthone from *Garcinia mangostana* and its cytotoxic activity. Nat. Prod. Res..

[32] Watanabe M., Gangitano E., Francomano D., Addessi E., Toscano R., Costantini D., Tuccinardi D., Mariani S., Basciani S., Spera G. (2018). Mangosteen extract shows a potent insulin sensitizing effect in obese female patients: A prospective randomized controlled pilot study. Nutrients.

[33] Ibrahim S.R.M., El-Agamy D.S., Abdallah H.M., Ahmed N., Elkablawy M.A., Mohamed G.A. (2018). Protective activity of tovophyllin A, a xanthone isolated from *Garcinia mangostana* pericarps, against acetaminophen-induced liver damage: Role of Nrf2 activation. Food Funct..

[34] Ibrahim S.R.M., Mohamed G.A., Elfaky M.A., Al Haidari R.A., Zayed M.F., El-Kholy A.A., Ross S.A. (2019). Garcixanthone A, a new cytotoxic xanthone from the pericarps of *Garcinia mangostana*. J. Asian Nat. Prod. Res..

[35] Mohamed G.A., Alliuocide G. (2008). A new flavonoid with potent α-amylase inhibitory activity from *Allium cepa* L.. Arkivoc.

[36] https://www.thermofisher.com/order/catalog/product/E33651?SID=srch-srp-E33651.

[37] Qian M., Spinelli S., Driguez H., Payan F. (2008). Structure of a pancreatic a-amylase bound to a substrate analogue at 2.03 a resolution. Protein Sci..

[38] Proença C., Freitas M., Ribeiro D., Tomé S.M., Oliveira E.F.T., Viegas M.F., Araújo A.N., Ramos M.J., Silva A.M.S., Fernandes P.A. (2019). Evaluation of a flavonoids library for inhibition of pancreatic α-amylase towards a structure-activity relationship. J. Enzyme Inhib. Med. Chem..

[39] Dandekar P.D., Kotmale A.S., Chavan S.R., Kadlag P.P., Sawant S.V., Dhavale D.D., RaviKumar A. (2021). Insights into the inhibition mechanism of human pancreatic α-amylase, a type 2 diabetes target, by dehydrodieugenol B isolated from *Ocimum tenuiflorum*. ACS Omega.

[40] Schrödinger, LLC. (2021). Schrödinger Release 2021-4: LigPrep.

[41] Omar A.M., Mohamed G.A., Ibrahim S.R.M. (2022). Chaetomugilins and chaetoviridins-promising natural metabolites: Structures, separation, characterization, biosynthesis, bioactivities, molecular docking, and molecular dynamics. J. Fungi.

[42] Schrödinger, LLC. (2021). Schrödinger Release 2021-4: Desmond Molecular Dynamics System.

[43] Ibrahim S.R.M., Omar A.M., Bagalagel A.A., Diri R.M., Noor A.O., Almasri D.M., Mohamed S.G.A., Mohamed G.A. (2022). Thiophenes-naturally occurring plant metabolites: Biological activities and in silico evaluation of their potential as cathepsin D inhibitors. Plants.

[44] Ferrari J., Terreaux C., Sahpaz S., Msonthi J.D., Wolfender J., Hostettmann K. (2000). Benzophenone glycosides from *Gnidia involucrata*. Phytochemistry.

[45] Kitanov G.M., Nedialkov P.T. (2001). Benzophenone O-glucoside, a biogenic precursor of 1,3,7-trioxygenated xanthones in Hypericum annulatum. Phytochemistry.

[46] Agrawal P.K. (1992). NMR spectroscopy in the structural elucidation of oligosaccharides and glycosides. Phytochemistry.

[47] Iwo M.I., Soemardji A.A., Hanafi M. (2013). Sunscreen activity of α-mangostin from the pericarps of *Garcinia mangostana* Linn. J. Appl. Pharm. Sci..

[48] Sen A.K., Sarkar K.K., Mazumder P.C., Banerji N., Uusvuori R., Hase T.A. (1982). The structures of garcinones A, B and C: Three new xanthones from *Garcinia mangostana*. Phytochemistry.

[49] Govindachari T.R., Kalyanaraman P.S., Muthukumaraswamy N., Pai B.R. (1971). Xanthones of *Garcinia mangostana* Linn. Tetrahedron.

[50] Xu Z., Huang L., Chen X.H., Zhu X.F., Qian X.J., Feng G.K., Lan W.J., Li H.J. (2014). Cytotoxic prenylated xanthones from the pericarps of *Garcinia mangostana*. Molecules.

[51] Osman A.G., Ali Z., Fantoukh O., Raman V., Kamdem R.S., Khan I. (2020). Glycosides of ursane-type triterpenoid, benzophenone, and iridoid from *Vangueria agrestis* (*Fadogia agrestis*) and their anti-infective activities. Nat. Prod. Res..

[52] Liu B., Chen N., Xu Y., Zhang J.W., Sun Y., Zhao L.Z., Ji Y.B. (2021). A new benzophenone with biological activities from metabolites of butyrolactone I in rat faeces. Nat. Prod. Res..

[53] Venditti A., Ukwueze S.E. (2017). A possible glycosidic benzophenone with full substitution on B-ring from *Psidium guajava* leaves. Nat. Prod. Res..

[54] Costa J.S., de Almeida A.A.C., Ferraz A.D.B.F., Rossatto R.R., Silva T.G., Silva P.B., Militão G.C., das Graças Lopes Citó A.M., Santana L.C., de Amorim Carvalho F.A. (2013). Cytotoxic and leishmanicidal properties of garcinielliptone FC, a prenylated benzophenone from Platonia insignis. Nat. Prod. Res..

[55] Akoro S.M., Aiyelaagbe O.O., Onocha P.A., Gloer J.B. (2020). Gakolanone: A new benzophenone derivative from Garcinia kola Heckel stem-bark. Nat. Prod. Res..

[56] Brayer G.D., Luo Y., Withers S.G. (1995). The structure of human pancreatic alpha amylase at 1.8 A resolution and comparisons with related enzymes. Protein Sci..

[57] Brayer G.D., Sidhu G., Maurus R., Rydberg E.H., Braun C., Wang Y., Nguyen N.T., Overall C.M., Withers S.G. (2000). Subsite mapping of the human pancreatic alpha-amylase active site through structural, kinetic, and mutagenesis techniques. Biochemistry.

[58] Rydberg E.H., Sidhu G., Vo H.C., Hewitt J., Côte H.C., Wang Y., Numao S., MacGillivray R.T., Overall C.M., Brayer G.D. (1999). Cloning, mutagenesis, and structural analysis of human pancreatic alpha-amylase expressed in Pichia pastoris. Protein Sci..

[59] Sahoo C.R., Paidesetty S.K., Dehury B., Padhy R.N. (2020). Molecular dynamics and computational study of Mannich-based coumarin derivatives: Potent tyrosine kinase inhibitor. J. Biomol. Struct. Dyn..

